# A Rapid Method for Quantifying RNA and Phytohormones From a Small Amount of Plant Tissue

**DOI:** 10.3389/fpls.2020.605069

**Published:** 2020-11-19

**Authors:** Da Cao, Francois Barbier, Kaori Yoneyama, Christine A. Beveridge

**Affiliations:** ^1^School of Biological Sciences, The University of Queensland, Brisbane, QLD, Australia; ^2^Graduate School of Agriculture, Ehime University, Matsuyama, Japan

**Keywords:** phytohormone and RNA extraction, phytohormone quantification, cytokinin, gibberellin, auxin, abscisic acid, UPLC-MS/MS, shoot branching

## Abstract

Phytohormones are involved in most plant physiological processes and the quantification of endogenous phytohormone levels and related gene expressions is an important approach to studying phytohormone functions. However, the quantification of phytohormones is still challenging due to their extremely low endogenous level in plant tissues and their high chemical diversity. Therefore, developing a method to simultaneously quantify phytohormone levels and RNA would strongly facilitate comparative analyses of phytohormones and gene expression. The present work reports a convenient extraction protocol enabling multivariate analysis of phytohormones and RNA from small amounts of plant material (around 10 mg). This high-throughput ultra-performance liquid chromatography-tandem mass spectrometry (UPLC-MS/MS) method demonstrates quantification of phytohormones and their related metabolites from four plant hormone classes: cytokinin, auxin, abscisic acid, and gibberellin. The UPLC-MS/MS method can quantify thirteen phytohormones and their metabolites simultaneously in 14 min. To validate the developed method, we determined the dynamic profiles of phytohormones and gene expressions in small axillary shoot buds in garden pea. This new method is applicable to quantification analysis of gene expression and multiple phytohormone classes in small amounts of plant materials. The results obtained using this method in axillary buds provide a basis for understanding the phytohormone functions in shoot branching regulation.

## Introduction

Phytohormones are endogenous signaling molecules that are involved in an immensely diverse range of plant physiological and developmental processes, which makes them critical for survival in most plant species. Despite their importance for plant growth regulation, not all phytohormones can yet be easily detected and quantified, which limits the speed of phytohormone related research significantly (Cao et al., 2017; Liu et al., 2019). Due to various chemical classes and ultra-trace amounts of phytohormones in plant tissues, it is difficult to measure phytohormones using a single separation method and analytical platform (Novák et al., 2017). Moreover, phytohormone levels vary between different plant tissues (Novák et al., 2017). Thus, it is important to select appropriate pre-treatment and quantification methods to boost the measurement sensitivity of targeted phytohormones (Yu et al., 2018).

Ultra-performance liquid chromatography-tandem mass spectrometry (UPLC-MS/MS) has become the most efficient method for boosting measurement sensitivity, as it provides high selectivity and sensitivity for phytohormone profiling (Pan et al., 2010; Schäfer et al., 2016; Šimura et al., 2018). However, mass spectrometry sensitivity is strongly influenced by other compounds in plant materials which suppress the ionization of target compounds (Trapp et al., 2014). Thus, a specific clean-up extraction method for phytohormones is needed. To quantify multiple phytohormone classes, many studies have used a time consuming parallel extraction method for different classes of phytohormone (Cao et al., 2016; Xin et al., 2020). In addition to phytohormone profiling, monitoring gene expression is a key requirement for understanding the involvement of phytohormones in plant physiology and development (Šimura et al., 2018; Barbier et al., 2019b). However, simultaneous extraction method of a wide range of phytohormones and RNA using one simple extraction method has not been reported.

Plant shoot branching is a perfect example of a developmental process involving multiple phytohormones. Auxins, cytokinins (CKs) and strigolactones (SLs) have been found to play major roles in shoot branching (reviewed by Barbier et al., 2019b). Auxin is mainly produced from the shoot tip and travels basipetally within the main stem to inhibit axillary bud outgrowth. Auxin can inhibit CK and promote SL biosynthesis gene expressions to regulate shoot branching (Ferguson and Beveridge, 2009). CK transports acropetally in xylem and synthesizes locally to promote shoot branching. SL, a new phytohormone, also transports acropetally from root to shoot but it inhibits shoot branching (Gomez-Roldan et al., 2008; Xie et al., 2016). It has also recently been proved that abscisic acid (ABA) and gibberellic acid (GA) are involved in shoot branching (Yao and Scott, 2015; Charnikhova et al., 2017). ABA signaling has been reported to inhibit shoot branching (González-Grandío et al., 2017). That GA biosynthesis mutants show branching phenotypes in many plant species, suggests that GA is implicated in shoot branching regulation (Lo et al., 2008; Martínez-Bello et al., 2015).

In the present study, we established an efficient quantitative method for quantifying 13 phytohormones and their related metabolites using UPLC-MS/MS. This technique was coupled with an integrative extraction method for phytohormones and RNA that provided a sensitive and convenient approach for phytohormone and gene expression quantification within the same sample. Using this method, we profiled hormone levels and quantified gene expressions in dormant and released axillary buds in garden pea.

## Materials and Methods

### Chemicals and Reagents

Phytohormone standards and internal standards (ISTD) used for the UPLC-MS/MS method were sourced from different suppliers (Supplementary Table 1). All solvents used were LC-MS grade purchased form Merck (Australia).

### Plant Growth and Harvest

Garden pea (*Pisum sativum*) plants were grown one per 68 mm square pot (width: 67 mm top, 47 mm bottom, and height: 96 mm) containing UQ23 potting mix with Osmocote fertilizers. Flowfeed EX7 (Grow Force) was supplied weekly. Plants were grown in a temperature controlled room with 23°C day/18°C night, and 18-h photoperiod. Two-week-old plants with five fully-expended leaves were used for sampling. Nodes were numbered acropetally from the first scale leaf as node 1. Decapitation was performed 1 cm above node 5. Node 2 buds were harvested after 24 h decapitation treatment. Twenty individual axillary buds were harvested and pooled as one biological replicate for hormone profiling and gene expression analysis. All samples were immediately snap frozen using liquid nitrogen and stored at −80°C until sample extraction.

### Simultaneous Extraction of Phytohormones and RNA in Acetonitrile

Four biological replicates were used for phytohormone and RNA extraction. The hormone extraction method was modified from Cao et al. (2017). Harvested samples were homogenized using 2010 Geno Grinder (SPEX SamplePrep, Metuchen, NJ, United States) under 4 C (1,500 rpm, 2 min × 1 min). Then, the extraction solvent was added with 1 mL 80% acetonitrile (ACN) containing 1% acetic acid (AcOH) and 5 μl ISTD working solution (10 ng mL^–1^ d_5_-*trans*-zeatin, 20 ng mL^–1^ d_3_-dihydrozeatin, 40 ng mL^–1^ d_5_-*trans*-zeatin-riboside, 100 ng mL^–1^ d_3_-dihydrozeatin riboside, 120 ng mL^–1^ d_5_-zeatin riboside-5′-monophosphate, 40 ng mL^–1^ d_6_-isopentenyladenine, 60 ng mL^–1^ d_6_-isopentenyladenosine, 10 ng mL^–1^ d_6_-*N*^6^-isopentenyl-adenosine-5′-monophosphate, 200 ng mL^–1^ d_5_-indole-3-acetic acid, 200 ng mL^–1^ d_2_-gibberellin A_1_, 200 ng mL^–1^ d_2_-gibberellin A_20_, 200 ng mL^–1^ d_2_-gibberellin A_29_ and 200 ng mL^–1^ d_6_-abscisic acid). After that, samples were left at −20°C for 5 min. Then, samples were centrifuged at 15,900 rcf at 4°C for 10 min. The phytohormones were extracted into the supernatant whereas the RNA was retained in the plant debris pellet.

#### Phytohormone Extraction

**FIGURE 1:**
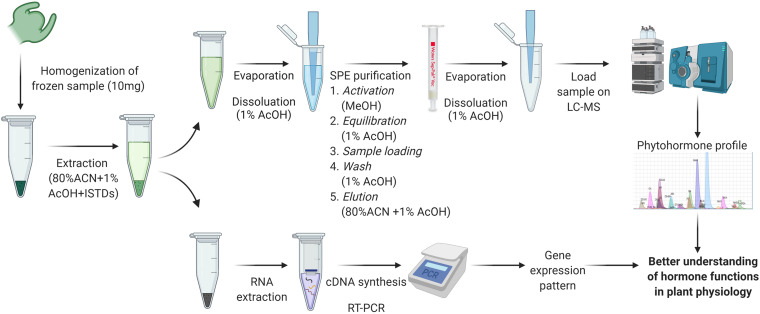
Extraction and quantification method workflow for phytohormones and RNA. Created with BioRender.com. ACN, acetonitrile; AcOH, acetic acid; ISTDs, internal standards; SPE, solid phase extraction.

The supernatant was transferred to a new 1.5 mL Eppendorf tube and dried using a rotational vacuum concentrator for hormone extraction. The dried extract was reconstituted in 1 mL of 1% AcOH and further purified with a Sep-Pak tC18 cartridge (Waters, United States). The tC18 cartridge was washed by 1 mL methanol, which was followed by an equilibration step with 1 mL 1% AcOH. The sample was loaded into the cartridge, and this was followed with a wash of 1 mL 1% AcOH. The extract was eluted with 1 mL 80% ACN containing 1% AcOH into a new 1.5 mL Eppendorf tube. The eluted extract was dried using a rotational vacuum concentrator and reconstituted in 20 μL 1% AcOH. The extract was vortexed for 30 s until the dried extract dissolved and then was centrifuged at 15,900 rcf at 4°C for 10 min. Samples were transferred to vials with a glass insert (Agilent, United States) and stored at −80°C until UPLC-MS/MS analysis (Figure 1).

#### RNA Extraction

Total RNA was extracted from the plant debris pellet using two RNA extraction methods. The first extraction method was undertaken using a Nucleospin RNA plant kit (Macherey Nagel, Bethlehem, PA, United States). The extraction protocol was followed as published online^1^. The second extraction was achieved using the CTAB-based method, during which the samples were extracted as in Barbier et al. (2019a). Briefly, 625 μL of CTAB buffer and 25 μL of DTT (0.5 M of stock solution) were added to each plant debris pellet. The samples were incubated for 10–15 min at 50–60°C and vortexed regularly. A similar volume of isopropanol was added to each sample. After vortexing and incubating the samples at −20°C for 15 min, they were centrifuged at 20,000 × *g* for 30 min. The supernatant was then discarded, and the pellet was washed with 70% ethanol and centrifuged at 20,000 × *g* for 10 min. The nucleic acid pellets were resuspended in RNase-free water and a DNase treatment was applied for 20 min. An equal volume of isopropanol was added to each sample. After vortexing and incubating the samples at −20°C for 15 min, they were centrifuged at 20,000 × *g* for 30 min. The supernatant was then discarded, and the pellet was washed with 70% ethanol and centrifuged at 20,000 × *g* for 10 min. The RNA pellets were resuspended in RNase-free water. The quality and concentration of extracted RNA were determined by NanoVue plus (GE Healthcare) and agarose gel electrophoresis.

### Phytohormone Profiling

#### Calibration Standard Sample Preparation

Thirteen phytohormone and related metabolite standards were used for method development (Supplementary Table 1). Standard stock solutions were prepared at 100 μg mL^–1^ and working solutions at 1 μg mL^–1^ in methanol. To prepare the calibration standard sample, phytohormone working solutions were mixed and serially diluted with starting mobile phase [0.5% formic acid (FA) in MilliQ water] to build up the calibration ranges at 0, 0.1, 0.5, 1, 5, 10, and 100 ng mL^–1^. The same ISTD concentration was added as for the phytohormone extraction.

#### Liquid Chromatography-Mass Spectrometry

The UPLC-MS/MS system was a Nexera X2 ultra high pressure liquid chromatograph (UPLC) system (Shimadzu Corporation, Japan) coupled with a 5,500 triple quadrupole linear ion trap (QTRAP) MS system equipped with an electrospray ionization source (ESI) (AB Sciex, United States). Kinetex C18 reversed phase UPLC column (2.1 mm × 100 mm, 1.7 mm) was used for phytohormone profiling. The optimized LC method was as follows: mobile phase A: 0.5% FA in MilliQ water (v/v); mobile phase B: 0.5% FA in ACN (v/v). Flow rate: 0.5 mL min^–1^. The programmed step gradient was: 4% B over 0.5 min, 4 to 15% B over 7 min, 15 to 95% B over 3.5 min, followed by a clean-up step: 95 to 95% B over 2 min, 95 to 4% B over 0.1 min and column re-equilibration for 1 min. ESI parameters (for positive and negative modes, respectively): curtain gas: 20 psi; collision gas: medium; ion source temperature: 500°C; ion source gas 1 and 2: 80 psi; Ion Spray voltage: +4,500 V, −4,500 V. The sMRM parameters are listed in Table 1.

**TABLE 1:** Scheduled multiple reaction monitoring (sMRM) parameters for phytohormone standards and their corresponding internal standards. CE, DP, and EP are the same between pH and ISTD.

PH	Q1	Q3	RT	SM	ISTD	Q1	Q3	RT	SM	CE	DP	EP
*t*Zeatin	220	136	2.3	+	d_5_-*t*z	225	136	2.2	+	25	75	10
DHZ	222	136	2.5	+	d_3_-DZ	225	136	2.4	+	25	100	8
*t*ZR	352	220	3.5	+	d_5_-*t*ZR	357	225	3.3	+	25	80	10
DHZR	354	222	3.6	+	d_3_-DZR	357	225	3.4	+	30	80	10
*t*ZMP	432	220	1.9	+	d_5_-*t*ZRP	437	225	1.8	+	25	100	10
iP	204	136	5.4	+	d_6_-iP	210	136	5	+	20	70	10
iPA	336	136	6.9	+	d_6_-iPA	342	136	6.5	+	40	80	10
iPAMP	416	136	4	+	d_6_-iPAMP	422	136	3.5	+	42	100	10
IAA	176	130	8.1	+	d_5_-IAA	181	135	8.1	+	25	120	15
GA_1_	347	229	7.6	−	d_2_-GA_1_	349	231	7.6	−	−40	−80	−15
GA_20_	331	287	9.5	−	d_2_-GA_20_	333	289	9.5	−	−30	−80	−15
GA_29_	347	303	4.8	−	d_2_-GA_29_	349	305	4.8	−	−30	−80	−15
ABA	263	153	9.4	−	d_6_-ABA	269	159	9.4	−	−20	−80	−15

*pH, phytohormone; ISTD, internal standard; Q1, precursor ion selected in Q1; Q3, product ion selected in Q3; CE, collision energy; RT, retention time; SM, scan mode; DP, declustering potential; EP, entrance potential; *t*Zeatin, *trans*-zeatin; *t*ZMP, *trans*-zeatin riboside-5′-monophosphate; DHZ, dihydrozeatin; *t*ZR, *trans*-zeatin riboside; DHZR, dihydrozeatin riboside; iPAMP, isopentenyladenosine-5′-Monophosphate; iP, isopentenyladenine; iPA, isopentenyladenosine; ABA, abscisic acid; GA_1,20_ and _29_, gibberellin A_1_, A_20_, and A_29_; IAA, indole-3-acetic acid.*

### RT-qPCR

cDNA synthesis and RT-qPCR were processed as described by Barbier et al. (2019a). Briefly, reverse transcription of 500 ng of RNA was performed using the iScript Supermix (Bio-Rad, United States). The cDNA was then diluted for quantitative RT-PCR. Quantitative RT-PCR was performed using SensiFAST^TM^ SYBR^®^ No-ROX Kit (Bioline) on a CFX384 Touch real-time PCR detection system (Bio-Rad).

### Data Processing and Statistical Analysis

Raw phytohormone data generated from LC-MS was analyzed using MultiQuant software (AB Sciex, United States). Raw RT-qPCR data was analyzed using CFX Manager 2.1 software (Bio-Rad) and LinRegPCR. Phytohormone levels in tested samples were quantified based on the peak area ratios of endogenous to corresponding ISTD compounds and the standard/ISTD ratios calculated with calibration curves as descripted in Delatorre et al. (2017). Graphpad prism 8.0 (Graphpad Software, United States) was used for statistical analysis. Statistical significance between experimental groups was performed using Student’s *t*-test.

## Results and Discussion

### Method Optimization

The integrative extraction method for phytohormones and RNA and the UPLC-MS/MS quantification method were optimized separately. For hormone and RNA extraction, the method was adapted from previous methods and optimized using available hormone standards. For UPLC-MS/MS, several important factors were optimized for enhancing system selectivity and sensitivity, including mobile phase, UPLC column and MS instrument parameters.

#### RNA Extraction

**FIGURE 2:**
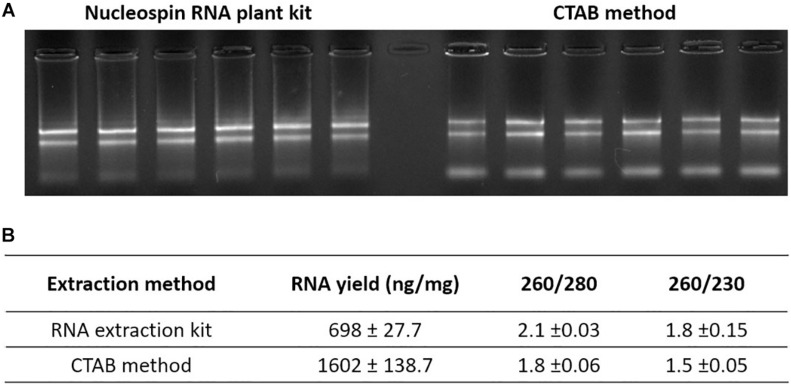
RNA extracted from hormone extraction pellet. **(A)** RNA gel of extracted pea axillary bud RNA with two RNA extraction methods. **(B)** Average RNA yield and purity measured using NanoVue. Values are mean ± SE, *n* = 6.

Acetonitrile (ACN) was chosen as the extraction solvent for phytohormones and RNA. Due to the low solubility of nucleic acids in organic solvents, RNA remained in the pellet while phytohormones from plant material dissolved in 80% ACN. Additionally, at this concentration, the ACN unfolds proteins, preventing RNase activity. To optimize the RNA precipitation, the extract was left at −20 C for 5 min. After centrifugation, the RNA left in the pellet was extracted using two different methods to test the RNA quality. The first method was a commercial silica column-based RNA extraction kit (Nucleospin RNA plant kit; Macherey-Nagel) and the second was a phenol/chloroform-free CTAB-based method (Barbier et al., 2019a). Although the CTAB based method takes slightly longer than the column-based method to perform, the CTAB method is cheaper and allows efficient extraction of RNA, including miRNA, from a very small amount of tissue, even in recalcitrant species (Barbier et al., 2019a). As demonstrated in Figure 2, good quality RNA was obtained using both methods. However, the RNA yield was higher when the CTAB method was used (Figure 2).

#### Phytohormone Extraction

For phytohormone extraction, extraction with blank control was performed to avoid contamination from lab materials. The pipette tips (Interpath Services, Heidelberg West, VIC, Australia) showed strong signs of contamination or leaching of a molecular signal identical to the phytohormone, isopentenyladenosine (MRM and retention time), one of the targets of this method (Supplementary Figure 2). Thus, we changed to another pipette tip brand (Vertex pipette tips, SSIbio, United States), which showed no contamination for any of our target compounds (Supplementary Figure 2). Moreover, to optimize the purity of the extracted phytohormones from the supernatant, a further clean-up step with Sep-Pak tC18 (Waters, United States) solid phase extraction (SPE) column was undertaken (Fu et al., 2011; Balcke et al., 2012).

#### UPLC-MS/MS

To develop the MS method, precursor identification of each phytohormone was conducted in Q3 MS scan mode using standard working solutions with direct injection (Supplementary Table 1). Identified precursor masses were confirmed with theoretical adduct masses. Fragment ion identification was conducted in product ion scan mode. Collision energy, declustering potential, entrance potential and ion source parameters were optimized using Analyst (AB Sciex, United States). These optimized parameters were used to develop the multiple reaction monitoring (MRM) method (Table 1).

The UPLC system was optimized using a mixture of phytohormone and ISTD working standards. Four mobile phase types with different ionization agents were compared, based on previous publications (Foo et al., 2007; Barbier et al., 2015; Cao et al., 2017). The first mobile phase tested contained ACN + 0.5% formic acid (FA); the second contained ACN + 10 mM ammonium acetate (NH4Ac); the third contained methanol + 0.5% FA; and the fourth contained ACN + 0.1% FA. The mobile phase containing ACN + 0.5% FA was chosen as the ideal mobile phase with best separation ability and MS sensitivity. Interestingly, we found that increasing FA concentration from 0.1 to 0.5% FA in the mobile phase could not only increase the MS sensitivity for CKs, but also improved the peak shape for compounds containing a phosphate group. The high FA/low pH likely enhances the MS response by promoting the formation of (M + H)^+^, and by decreasing the interaction between the phosphate compound and stainless steel (Wakamatsu et al., 2005; Cao et al., 2016).

**FIGURE 3:**
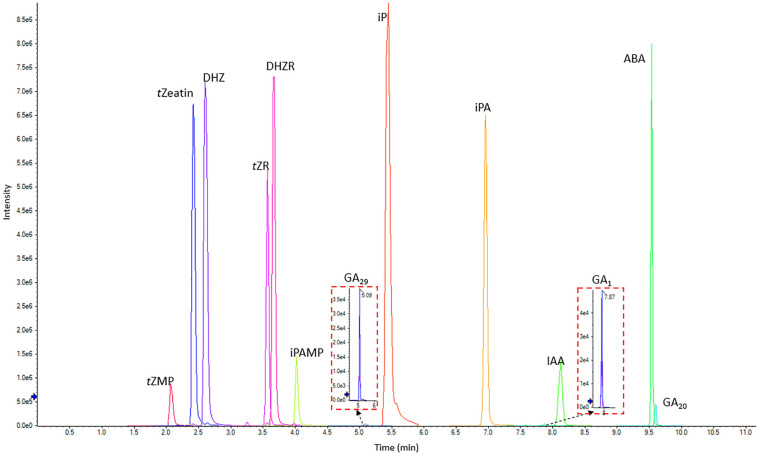
LC-MS chromatogram showing the separation of 13 phytohormone standards (0.5 μg/mL each) including CKs, IAA, ABA, and GAs. *t*Zeatin, *trans*-zeatin; *t*ZMP, *trans*-zeatin riboside-5′-monophosphate; DHZ, dihydrozeatin; *t*ZR, *trans*-zeatin riboside; DHZR, dihydrozeatin riboside; iPAMP, isopentenyladenosine-5′-Monophosphate; iP, isopentenyladenine; iPA, isopentenyladenosine; ABA, abscisic acid; GA_1,20_ and _29_, gibberellin A_1_, A_20_, and A_29_; IAA, indole-3-acetic acid.

C18 columns have been widely used for phytohormone quantification but with some disadvantages, such as instability in aqueous solutions and relatively low pH stability (Pan et al., 2010; Liu et al., 2019; Xin et al., 2020). Thus, two types of UPLC C18 columns were compared based on their pH and aqueous stability. We tested the normal C18 column, Kinetex C18 reversed phase column (2.1 mm × 100 mm, 1.7 μm) and Kinetex EVO C18 reversed phase column (2.1 mm × 100 mm, 1.7 μm), which is designed with higher aqueous and pH stability^2^. However, the EVO C18 column showed inefficient separation ability for two pairs of hydrophilic phytohormone compounds (Supplementary Figure 1). Thus, the Kinetex C18 reversed phase column was chosen as the ideal column for phytohormone profiling for the present method. The optimized UPLC and MS/MS methods were combined to build up the final scheduled MRM (sMRM) method (Figure 3).

### Method Validation

To validate the LC-MS method, limits of detection (LOD), limits of quantification (LOQ), calibration curve linearity, recovery and repeatability were tested using a pooled biological quality control (PBQC) tissue sample (Cao et al., 2017). The PBQC was a mixture of extracts from pea stem tissues. Three PBQCs were extracted individually and used for LOD, LOQ, and RSD% calculation. The LOD and LOQ were calculated based on the signal to noise ratio (S/N) of 3 and 10, respectively. The LOD for all phytohormones ranged from 0.01 to 1.8 ng g^–1^. The LOQ ranged from 0.08 to 5.9 ng g^–1^ (Table 2). Repeatability was calculated using the percent relative standard deviation (RSD%) (standard deviation/mean × 100%) (Kele and Guiochon, 1999; Shabir, 2004) and were all less than 8.8% (Table 2), which indicates good repeatability for our method. The linearity for each phytohormone was calculated using calibration standard samples with a 1/x^2^ weighting, and regression equations (*R*^2^) were greater than 0.98 across three orders of magnitudes (0, 0.1, 0.5, 1, 5, 10, and 100 ng mL^–1^). Recoveries for all phytohormones were calculated by spiking corresponding hormone standards into a separate PBQC (Pan et al., 2010) and ranged from 69 to 98% (Table 2).

**TABLE 2:** Limit of detection (LOD), limit of quantification (LOQ), repeatability, linearity, and recovery for developed phytohormone profiling method.

PH	LOD (ng g^–1^)	LOQ (ng g^–1^)	Repeatability (RSD%)	*R*^2^	Recovery (%)
*t*Zeatin	0.03	0.09	8.13	0.996	70
DHZ	0.02	0.08	7.71	0.994	77
*t*ZR	0.04	0.12	6.34	0.991	91
DHZR	0.05	0.17	2.41	0.987	75
*t*ZMP	0.17	0.55	6.06	0.999	75
iP	0.08	0.26	5.58	0.999	69
iPA	0.01	0.03	4.16	0.999	87
iPAMP	0.21	0.70	1.95	0.997	71
IAA	1.79	5.96	5.01	0.992	96
GA_1_	0.12	0.40	5.07	0.997	95
GA_20_	0.98	3.27	5.00	0.995	72
GA_29_	0.86	2.87	8.80	0.995	98
ABA	0.03	0.11	7.90	0.999	74

*PH, phytohormone; RSD%, relative standard deviation.*

### Phytohormone Responses With Decapitation Treatment

This method was demonstrated for the study of shoot branching which is regulated by several phytohormone signals. Most plant species exhibit a branching response with decapitation treatment, which is a shoot tip removal assay that breaks the apical dominance and induces the outgrowth of axillary buds (McSteen and Leyser, 2005; Barbier et al., 2019b). In the present study, we profiled phytohormone levels in axillary buds with decapitation treatment to study phytohormone functions on shoot branching.

**FIGURE 4:**
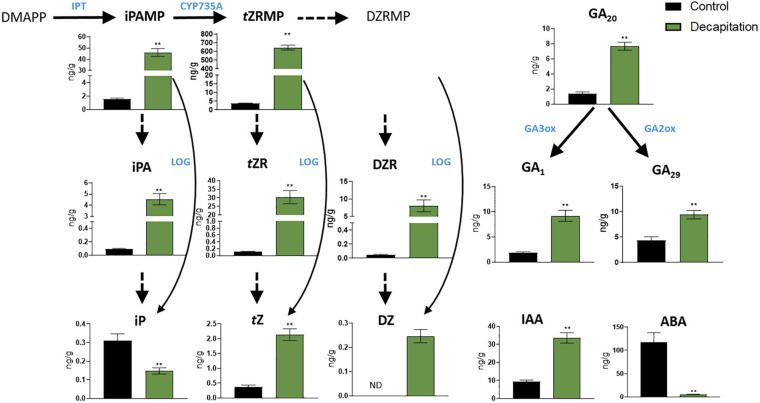
Phytohormone responses in node 2 axillary buds with decapitation treatment after 24 h. Values are mean ± SE, *n* = 4. **P < 0.01 compared with control, Student’s *t*-test. DMAPP, dimethylallyl diphosphate; iPAMP, isopentenyladenosine-5′-Monophosphate; *t*ZRMP, *trans*-zeatin riboside-5′-monophosphate; DZRMP, dihydrozeatin riboside-5′-monophosphate; iPA, isopentenyladenosine; *t*ZR, *trans*-zeatin riboside; DZR, dihydrozeatin riboside; iP, isopentenyladenine; *t*Z, *trans*-zeatin; DZ, dihydrozeatin; ABA, abscisic acid; GA_1, 20_ and _29_, gibberellin A_1_, A_20_, and A_29_; IAA, indole-3-acetic acid; GA3ox and GA2ox, gibberellin 3 and 2 β-hydroxylase; IPT, adenosine phosphate-isopentenyltransferase; LOG, cytokinin phosphoribohydrolase “LONELY GUY”; CYP735A, cytochrome P450 mono-oxygenases; ND, not detectable.

By using our established method, we quantified phytohormone levels in pea axillary buds after 24 h decapitation treatment (Figure 4). Thirteen phytohormones and related metabolites were detectable, including three bioactive CKs: isopentenyladenine, *trans*-zeatin, and dihydrozeatin, and five of their biosynthesis precursors; three types of GAs, including bioactive GA_1_ and its biosynthesis precursor GA_20_, and related metabolite GA_29_; IAA and ABA (Figure 4 and Supplementary Figure 4). The MS dataset has been deposited in MetaboLights (MTBLS2078). Levels of all CKs and related metabolites showed significant increases of (6 to 276-fold) in axillary buds after 24 h decapitation treatment, except for iP (decreased 1.4-fold). GAs (2 to 5-fold) and IAA (4-fold) levels also significantly increased while ABA (23-fold) levels significantly decreased in axillary buds.

Cytokinin responses in pea axillary buds after decapitation have been reported previously. Tanaka et al. (2006) profiled CK responses in axillary buds (about 100 mg) in garden pea after 3 and 6 h decapitation treatment, and again found that most levels of CKs and related metabolites other than iP increased after decapitation. With 10 times less plant material used, our results are highly consistent with those of Tanaka et al. (2006) in terms of CK concentrations in pea buds and of responses with decapitation treatment. This validates the high sensitivity and accuracy of our method and indicates that iP and zeatin-type CK levels in buds consistently differ in response to decapitation. Moreover, we found IAA levels increase and ABA levels decrease in axillary buds after decapitation treatment, which is in agreement with findings in other species (Gocal et al., 1991; Galoch et al., 1998). These results provide support for the hypothesis that IAA accumulation and ABA depletion in buds influences their outgrowth (Nguyen and Emery, 2017; Chabikwa et al., 2019). We also profiled GA concentration in axillary buds with decapitation treatment. The increase in GA level in buds is consistent with the hypothesis that auxin promotes GA biosynthesis in decapitated plants (Wolbang and Ross, 2001). Our results provide the first and most extensive phytohormone profiling in axillary buds with decapitation treatment, which adds important information and technical capacity for understanding phytohormone functions in shoot branching.

### Bud Dormancy Marker Responses With Decapitation Treatment

**FIGURE 5:**
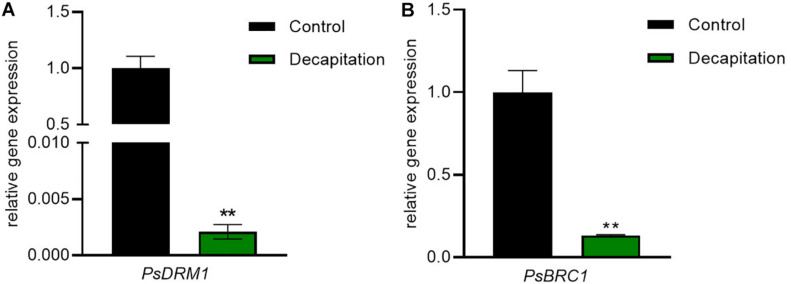
Bud dormancy marker gene responses in node 2 axillary buds with decapitation treatment after 24 h. **(A)** *PsDRM1* and **(B)** *PsBRC1* expressions in axillary buds were significantly decreased by decapitation treatment. Values are mean ± SE, *n* = 4. **P < 0.01 compared with control, Student’s *t*-test.

In order to test whether the RNA extracted with this method gives reliable results, we measured the expression of *BRC1* and *DRM1*, two markers of bud dormancy known to be inhibited by decapitation (Aguilar-Martínez et al., 2007; Fichtner et al., 2017; González-Grandío et al., 2017). In this study, we determined the transcriptional responses of *PsDRM1* and *PsBRC1* in the same sample used for hormone quantification. The extracted RNA can be successfully used for quantitative RT-PCR (Supplementary Figure 3). As expected, decapitation treatment strongly inhibited expression of *PsDRM1* (480-fold) and *PsBRC1* (7-fold) in axillary buds (Figure 5). These results are consistent with those of previous publications and demonstrate the success and feasibility of our integrative extraction method for RNA and phytohormones.

## Conclusion

We have presented a combined extraction method for phytohormones and RNA. This optimized method can extract and quantify RNA and phytohormones from the same sample using small sample amounts (10 mg). Except for axillary buds, this method has been tested and applied for pea stem tissues and *Arabidopsis* rosette tissues (data not shown). In only 14 min and with good reproducibility, the UPLC-MS/MS quantification method can measure 13 phytohormones using one single injection. These phytohormones include CKs, GAs, auxin, and ABA as well as some of their derivatives and precursor molecules. The optimized protocol provides a basis for understanding phytohormone functions in shoot branching regulation using experiments with pools, in the case of garden pea, of just twenty axillary buds and readily achieved with to 10 mg of tissue.

## Data Availability Statement

We have uploaded our mass spec dataset to MetaboLights. The study identifier is: MTBLS2078.

## Author Contributions

DC developed the LC-MS method and wrote the manuscript. DC and FB developed the extraction method. FB, KY, and CB critically reviewed the manuscript and advised on study design. All authors contributed to the article and approved the submitted version.

## Conflict of Interest

The authors declare that the research was conducted in the absence of any commercial or financial relationships that could be construed as a potential conflict of interest.

## Footnotes

1 https://www.mn-net.com/media/pdf/44/0d/83/Instruction-NucleoSpin-RNA-Plant.pdf

2 https://www.phenomenex.com/Info/Page/2014knxevo
