# Supplementary material for: A Rapid Method for Quantifying RNA and Phytohormones From a Small Amount of Plant Tissue
Source: Front Plant Sci. 2020 Nov 19;11:605069. doi: 10.3389/fpls.2020.605069 (PMC7717934; doi:10.3389/fpls.2020.605069)
Supplement: Supplementary file 1 [file Presentation_1.PPTX]

## Slide 1
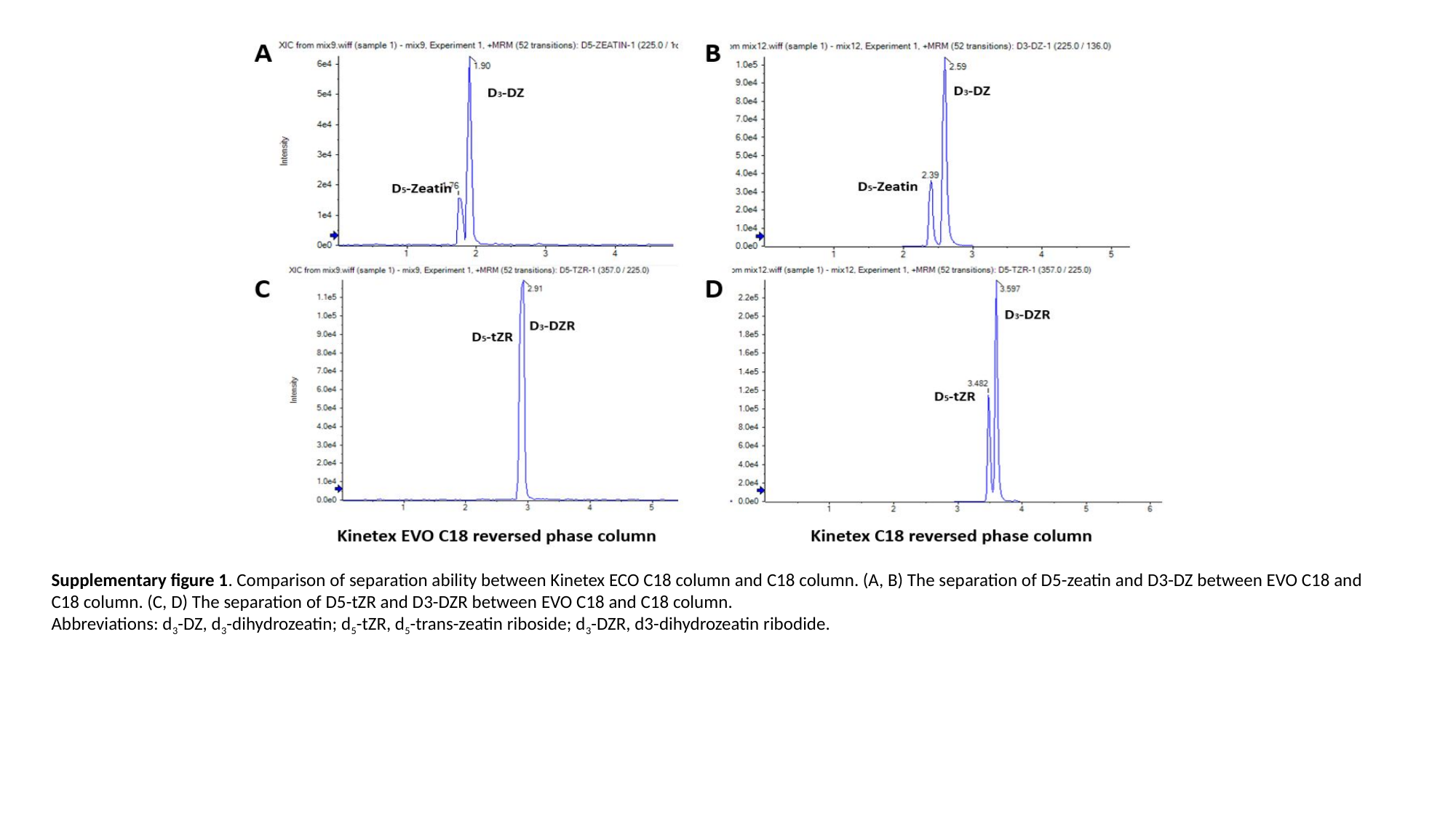

Supplementary figure 1. Comparison of separation ability between Kinetex ECO C18 column and C18 column. (A, B) The separation of D5-zeatin and D3-DZ between EVO C18 and C18 column. (C, D) The separation of D5-tZR and D3-DZR between EVO C18 and C18 column.
Abbreviations: d3-DZ, d3-dihydrozeatin; d5-tZR, d5-trans-zeatin riboside; d3-DZR, d3-dihydrozeatin ribodide.
