# Supplementary material for: A Rapid Method for Quantifying RNA and Phytohormones From a Small Amount of Plant Tissue
Source: Front Plant Sci. 2020 Nov 19;11:605069. doi: 10.3389/fpls.2020.605069 (PMC7717934; doi:10.3389/fpls.2020.605069)
Supplement: Supplementary file 2 [file Presentation_2.PPTX]

## Slide 1
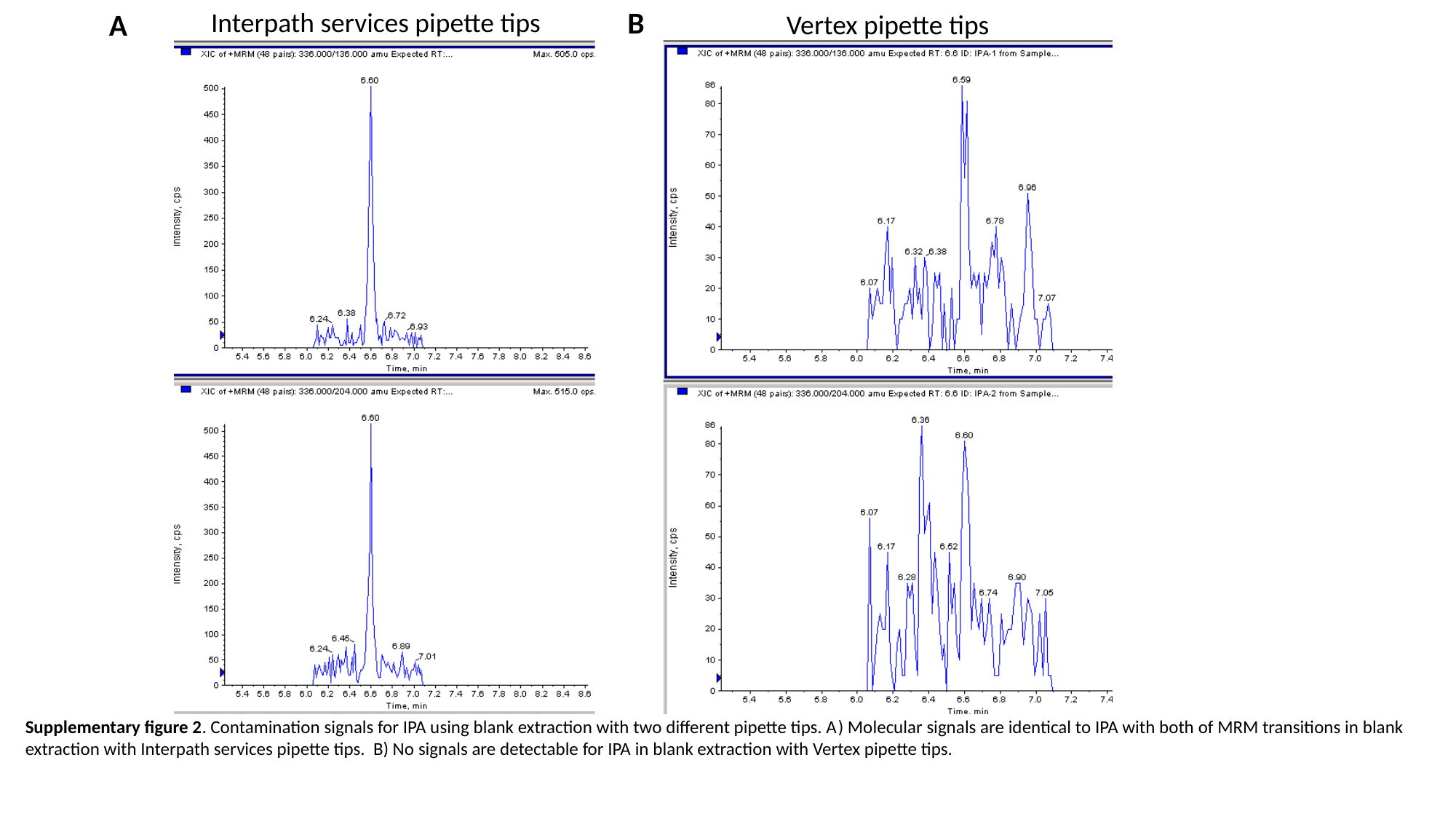

A
Interpath services pipette tips
B
Vertex pipette tips
Supplementary figure 2. Contamination signals for IPA using blank extraction with two different pipette tips. A) Molecular signals are identical to IPA with both of MRM transitions in blank extraction with Interpath services pipette tips. B) No signals are detectable for IPA in blank extraction with Vertex pipette tips.
