# Supplementary material for: A Rapid Method for Quantifying RNA and Phytohormones From a Small Amount of Plant Tissue
Source: Front Plant Sci. 2020 Nov 19;11:605069. doi: 10.3389/fpls.2020.605069 (PMC7717934; doi:10.3389/fpls.2020.605069)
Supplement: Supplementary file 3 [file Presentation_3.PPTX]

## Slide 1
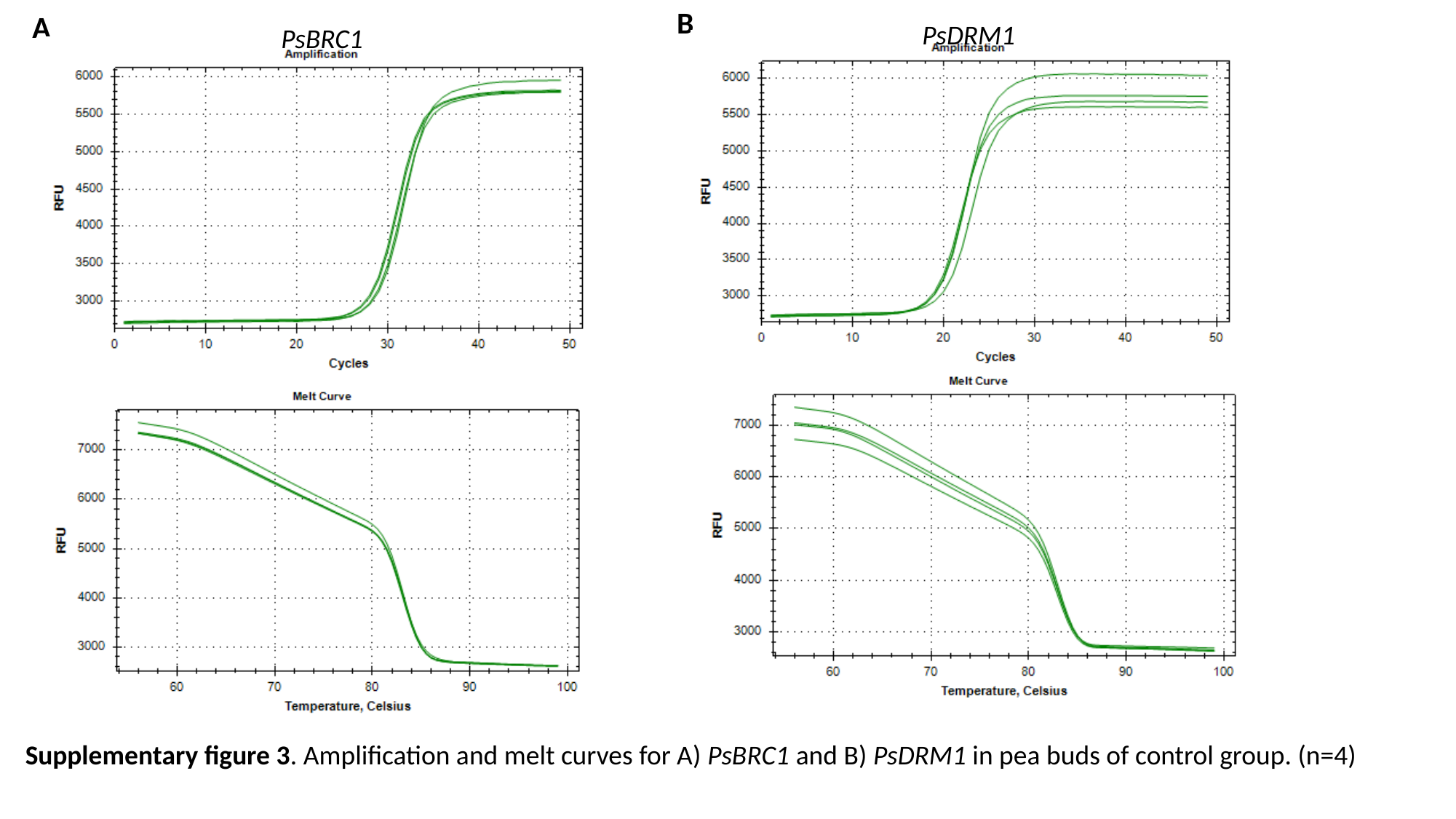

B
A
PsDRM1
PsBRC1
Supplementary figure 3. Amplification and melt curves for A) PsBRC1 and B) PsDRM1 in pea buds of control group. (n=4)
