# Supplementary material for: A Rapid Method for Quantifying RNA and Phytohormones From a Small Amount of Plant Tissue
Source: Front Plant Sci. 2020 Nov 19;11:605069. doi: 10.3389/fpls.2020.605069 (PMC7717934; doi:10.3389/fpls.2020.605069)
Supplement: Supplementary file 4 [file Presentation_4.PPTX]

## Slide 1
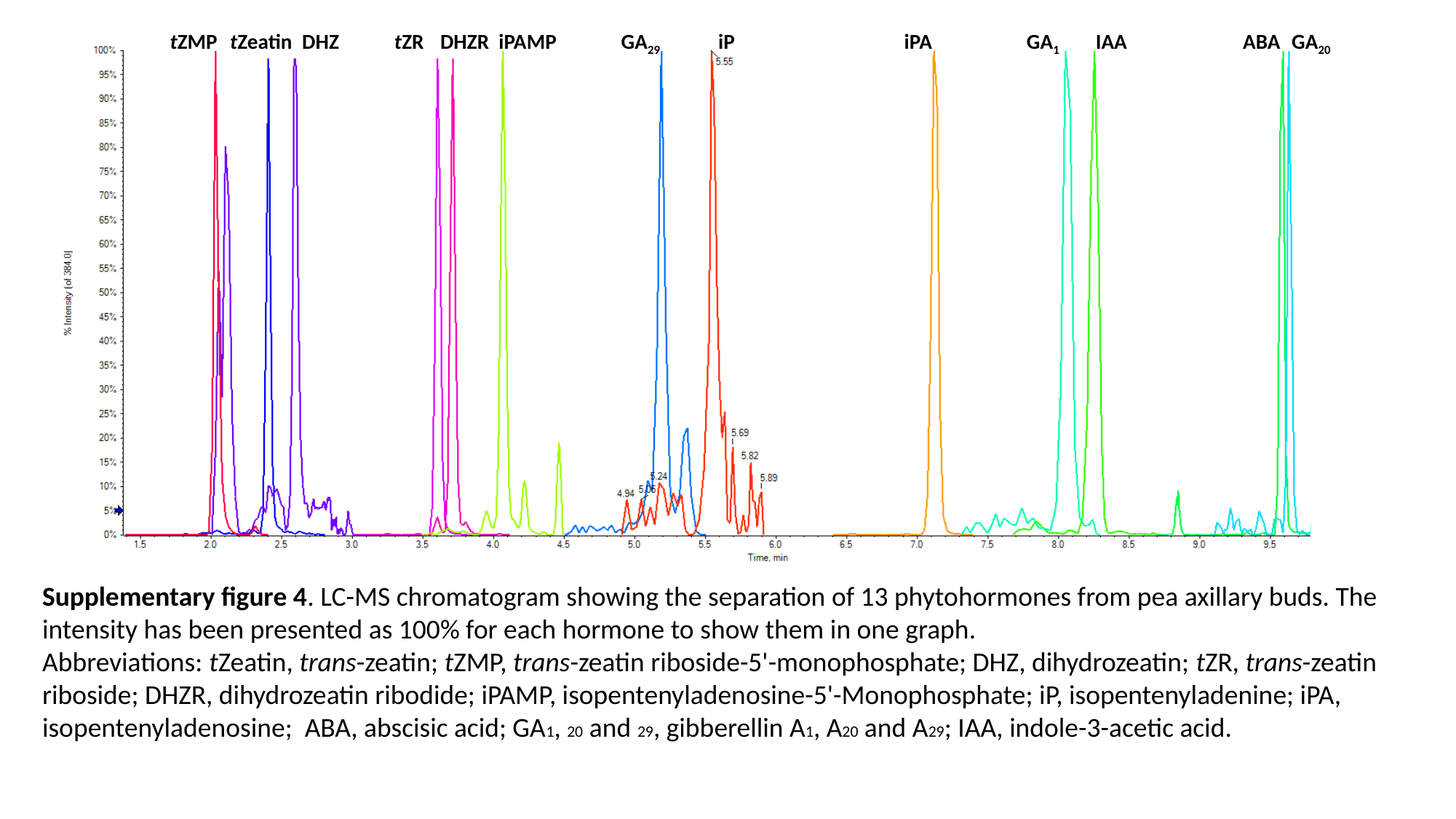

iPA
GA1
IAA
ABA
GA20
iP
iPAMP
GA29
tZMP
tZeatin
DHZ
tZR
DHZR
Supplementary figure 4. LC-MS chromatogram showing the separation of 13 phytohormones from pea axillary buds. The intensity has been presented as 100% for each hormone to show them in one graph.
Abbreviations: tZeatin, trans-zeatin; tZMP, trans-zeatin riboside-5'-monophosphate; DHZ, dihydrozeatin; tZR, trans-zeatin riboside; DHZR, dihydrozeatin ribodide; iPAMP, isopentenyladenosine-5'-Monophosphate; iP, isopentenyladenine; iPA, isopentenyladenosine; ABA, abscisic acid; GA1, 20 and 29, gibberellin A1, A20 and A29; IAA, indole-3-acetic acid.
